# Efficacy and safety of mazdutide vs. dulaglutide in adults with type 2 diabetes: a systematic review and meta-analysis of randomized controlled trials

**DOI:** 10.1186/s13098-026-02248-3

**Published:** 2026-07-24

**Authors:** Muhammad Hassan, Muhammad Hussain, Saifullah Khan, Muhammad Tayyab Azam, Zain Ul Abideen Shahid, Abdul Hannan, Muhammad Mussawir Khuhro, Sahil Bhagia, Hasibullah Aminpoor

**Affiliations:** 1 Dow University of Health Sciences, Karachi, Pakistan; 2 Faculty of Medicine, Kabul University of Medical Sciences “Abu Ali Ibn Sina”, Kabul, Afghanistan

## Abstract

**Background:**

Type 2 diabetes mellitus (T2DM) is closely linked to obesity and carries a high risk of metabolic complications. While GLP-1 receptor agonists like dulaglutide improve blood sugar and support modest weight loss, many patients do not reach their treatment goals. Mazdutide, a dual GLP-1 and glucagon receptor agonist, may offer stronger metabolic benefits.

**Objective:**

To compare the efficacy and safety of mazdutide versus dulaglutide in adults with T2DM.

**Methods:**

We systematically reviewed randomized controlled trials comparing mazdutide and dulaglutide. Primary outcomes included changes in HbA1c, body weight, and achievement of glycemic and weight-loss targets. Safety outcomes included treatment-emergent adverse events. Data were pooled using random-effects models, and evidence certainty was assessed using GRADE.

**Results:**

Three trials met inclusion criteria. Mazdutide led to greater reductions in HbA1c (− 0.25%) and body weight (− 2.97 kg) compared with dulaglutide. Patients were more likely to achieve HbA1c < 7.0%, ≥ 5% weight loss, and the combined target of ≥ 5% weight loss with HbA1c < 7.0%. Fasting plasma glucose was numerically but not significantly improved. Mazdutide was associated with higher rates of gastrointestinal side effects and decreased appetite, with a non-significant numerical increase in hypoglycemia, while serious adverse events and discontinuations were similar between treatments. Glycemic and major weight outcomes were supported by high-certainty evidence; most safety outcomes were moderate-certainty.

**Conclusions:**

Mazdutide, based on evidence from three RCTs, provides preliminary evidence of meaningful improvements in blood sugar control and weight loss compared with dulaglutide, though with an increased risk of mostly manageable gastrointestinal effects; given the limited number of trials, these findings should be considered preliminary and supporting its potential role as a promising dual-action therapy for T2DM management, pending confirmation in larger, longer-term trials.

**Graphical abstract:**

**Figure:**
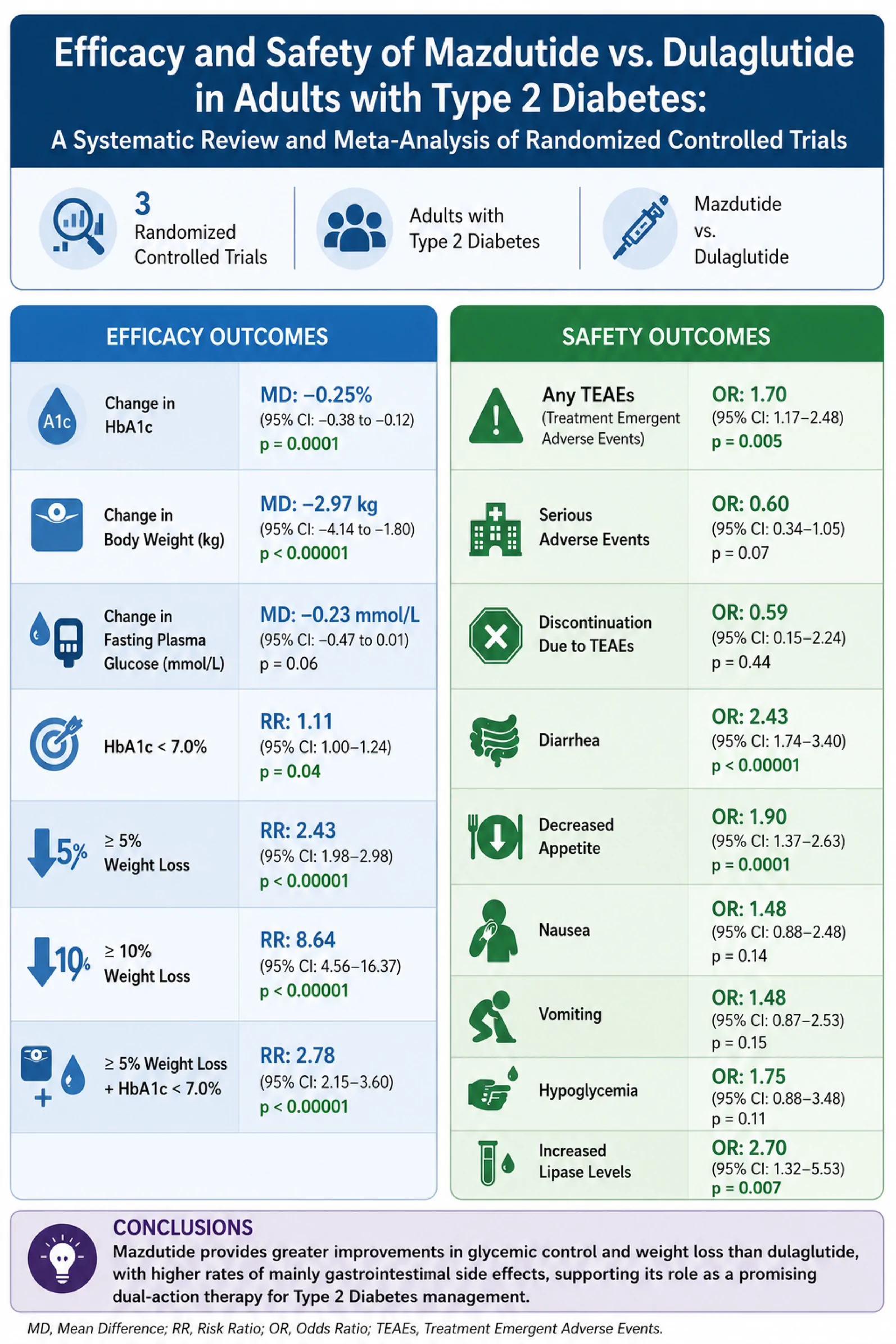

**Supplementary Information:**

The online version contains supplementary material available at https://doi.org/10.1186/s13098-026-02248-3.

## Introduction

Type 2 diabetes mellitus (T2DM) has become a rapidly escalating global health challenge, with its prevalence increasing in parallel with rising rates of overweight and obesity. More than 500 million adults globally are estimated to live with diabetes, and this number is projected to grow significantly over the coming decades as the obesity epidemic continues to expand worldwide [1]. Obesity, a major driver of insulin resistance and metabolic dysregulation, affects over 650 million adults and contributes substantially to the development and progression of T2DM, reflecting a dual burden of cardiometabolic disease [2, 3].

At its core, T2DM is characterized by chronic hyperglycemia due to impaired pancreatic β-cell function, peripheral insulin resistance, and dysregulation of hepatic glucose production [4, 5]. These metabolic abnormalities are frequently accompanied by dyslipidemia, adipose tissue dysfunction, and systemic inflammation, all of which contribute to an increased risk of microvascular and macrovascular complications, including cardiovascular disease and chronic kidney disease [6]. Contemporary management guidelines emphasize not only glycemic control but also reductions in excess body weight and comprehensive cardiometabolic risk reduction [7]. Despite the range of available glucose-lowering therapies, including oral antidiabetic drugs and insulin, many patients fail to achieve durable glycemic targets, and these treatments often have limited effects on weight and are associated with an increased risk of hypoglycemia [8]. Glucagon-like peptide-1 (GLP-1) receptor agonists have shifted the treatment paradigm by enhancing glucose-dependent insulin secretion, suppressing appetite, and promoting weight loss; meta-analyses have shown that GLP-1 receptor agonists can reduce glycated hemoglobin (HbA1c) by approximately 1.0–1.5% with concomitant clinically significant weight reduction and cardiovascular benefits [9–11]. Nonetheless, a considerable proportion of patients continue to experience suboptimal outcomes despite these therapies, highlighting an unmet need for more effective treatments [11].

To address these limitations, next-generation incretin-based co-agonists targeting multiple hormonal pathways have been developed. Dual receptor agonists that combine GLP-1 and glucagon activity are designed to complement the insulinotropic and anorectic effects of GLP-1 with glucagon-mediated increases in energy expenditure and lipid oxidation. This integrative mechanism has the potential to produce more robust metabolic benefits including improved glycemic control and weight loss [12, 13]. Mazdutide (IBI362/LY3305677) is a once-weekly synthetic analogue of oxyntomodulin functioning as a GLP-1 and glucagon receptor dual agonist. A phase 2 trials have demonstrated that mazdutide is well tolerated and produces clinically meaningful reductions in HbA1c and body weight, with more pronounced effects than those observed with dulaglutide in diabetic populations [14].

Increasingly compelling evidence has emerged from large, phase 3 trials. In the DREAMS-2 study (NCT05606913), mazdutide (4 mg and 6 mg) demonstrated superior reductions in mean HbA1c from baseline compared with dulaglutide 1.5 mg over 28 weeks, along with significantly greater weight loss and higher proportions of participants achieving clinically relevant glycemic and weight endpoints [15]. Moreover, the phase 3 DREAMS-3 head-to-head trial against semaglutide showed that mazdutide significantly outperformed semaglutide in combined glycemic and weight loss targets in adults with T2DM and obesity, reinforcing its clinical potential [16]. Despite this rapidly expanding evidence base, trial designs, comparator regimens, and outcome definitions vary considerably across studies, making it challenging to synthesize and interpret the overall magnitude and consistency of mazdutide’s benefits. In particular, the relative efficacy and safety of mazdutide versus dulaglutide a widely used GLP-1 receptor agonist have not yet been comprehensively evaluated across all relevant metabolic and safety outcomes. Therefore, a timely and rigorous systematic review and meta-analysis is urgently needed to integrate the current clinical evidence, clarify comparative advantages and risks, and guide evidence-based decision-making for adults with T2DM inadequately controlled on diet, exercise, or oral antidiabetic therapy.

## Method

We registered the protocol for this systematic review and meta-analysis with PROSPERO (CRD420261342203) [17] to ensure transparency and minimize potential methodological error. Throughout the study, we adhered to all the details prespecified in PROSPERO. The review was conducted in accordance with the PRISMA 2020 (Preferred Reporting Items for Systematic Reviews and Meta-Analyses) guidelines to uphold high standards of reporting and methodological rigor.

### Search strategy

A comprehensive search was conducted through CENTRAL-Cochrane Central Register of Controlled Trials, Embase.com, PubMed and Scopus to identify relevant randomized controlled trials (RCTs) comparing the efficacy of mazdutide and Dulaglutide in T2DM patients. The search covered studies from inception to March 16, 2026. Only articles published in English were included. Google Scholar was additionally searched to identify relevant gray literature. We utilized a combination of medical subject headings (MeSH), keywords and free-text terms (Supplementary Table 1).

### Eligibility criteria

Eligible studies included RCTs involving diabetic adults aged > 18 years with inadequate control on diet/exercise or stable oral anti-diabetic drugs. Participants in the intervention group received various doses of mazdutide, including 3 mg, 4 mg, 4.5 mg, 6 mg administered once-weekly subcutaneously, while the control group received Dulaglutide 1.5 mg once-weekly subcutaneously. Across all three included trials, dulaglutide 1.5 mg/week was the only comparator dose employed and consequently the only dose available for pooling in this meta-analysis. We excluded duplicates, conference abstracts and studies that did not report relevant outcomes. Two independent reviewers screened article titles and abstracts, followed by full-text reviews to assess eligibility. Any conflict between them was resolved through discussion with a third reviewer.

### Data extraction

Two independent reviewers extracted data from the included studies, focusing on key study details, including study name, first author, DOI, clinical trial registration number and treatment duration. Baseline characteristics, including sample size, gender ratio and treatment details (drug name and dosage), were also collected. To compare the efficacy of mazdutide and dulaglutide in T2DM patients, primary efficacy outcomes included Mean change in HbA₁c (%) from baseline to study end (week 28/20/12 as reported). Primary safety outcome: Incidence of treatment-emergent adverse events (TEAEs) i.e. diarrhoea, nausea and vomiting. Secondary outcomes included: Percentage change in body weight from baseline. Proportion achieving HbA₁c and lt;7.0% and/or ≥ 5% weight loss, change in fasting plasma glucose, discontinuations due to adverse events. Hypoglycemia was extracted as reported in the adverse event tables of each included trial. Classification criteria were explicitly defined in two of the three trials: the Zhang et al. trial applied the American Diabetes Association (ADA) 2013 classification of hypoglycemia, and the Guo et al. trial applied ADA 2022 criteria with explicit glucose-threshold tiers (Level 1, < 3.9 mmol/L; Level 2, < 3.0 mmol/L). The Jiang et al. trial did not report a specific classification scheme for hypoglycemia; events were captured only as a MedDRA preferred term with investigator-assessed severity (mild/moderate/severe), with no stated glucose threshold. Any disagreements during data extraction between the two reviewers were resolved through consultation with a third reviewer.

### Statistical analysis

A random-effects model was applied for the systematic review and meta-analysis using RevMan 5.4 [18]. Continuous outcomes were analyzed using mean differences (MDs) with 95% confidence intervals (CIs), and categorical outcomes using relative risk (RR) or Odds Ratio. Heterogeneity was assessed with the Chi-squared test and I2 statistics [19], with *p* < 0.05 considered significant. In trials with multiple mazdutide dose arms compared against a single shared dulaglutide control group, the comparator group was divided equally among the relevant comparisons to avoid double-counting and ensure independence of effect estimates, in accordance with Cochrane Handbook recommendations. Subgroup analyses were conducted by dosage and treatment duration, with differences tested using the chi-squared test for interaction and I2 statistics [19]. Sensitivity analyses used a leave-one-out approach to assess heterogeneity and the influence of individual studies. Publication bias was not assessed because fewer than 10 studies were available.

### Assessment of confidence in findings

The overall quality of the evidence was assessed using the GRADE (Grading of Recommendations, Assessment, Development, and Evaluation) approach [20]. This evaluation included five domains: risk of bias, inconsistency, indirectness, imprecision and publication bias. Evidence from RCTs was initially considered of high quality and downgraded if any concerns were identified in these domains. If a serious concern was found, the evidence was downgraded by one level; if very serious, by two levels. After evaluating all five domains, the overall confidence in the evidence was classified as high, moderate, low, or very low.

### Risk of bias assessment

The risk of bias of included randomized controlled trials was independently assessed by two reviewers using the Cochrane Risk of Bias 2 (RoB 2) tool [21]. The following domains were evaluated: bias arising from the randomization process, deviations from intended interventions, missing outcome data, measurement of outcomes, and selection of reported results. Each study was classified as having low risk, some concerns, or high risk of bias. Discrepancies between reviewers were resolved through discussion with a third reviewer.

## Results

### Study selection

**Fig. 1:**
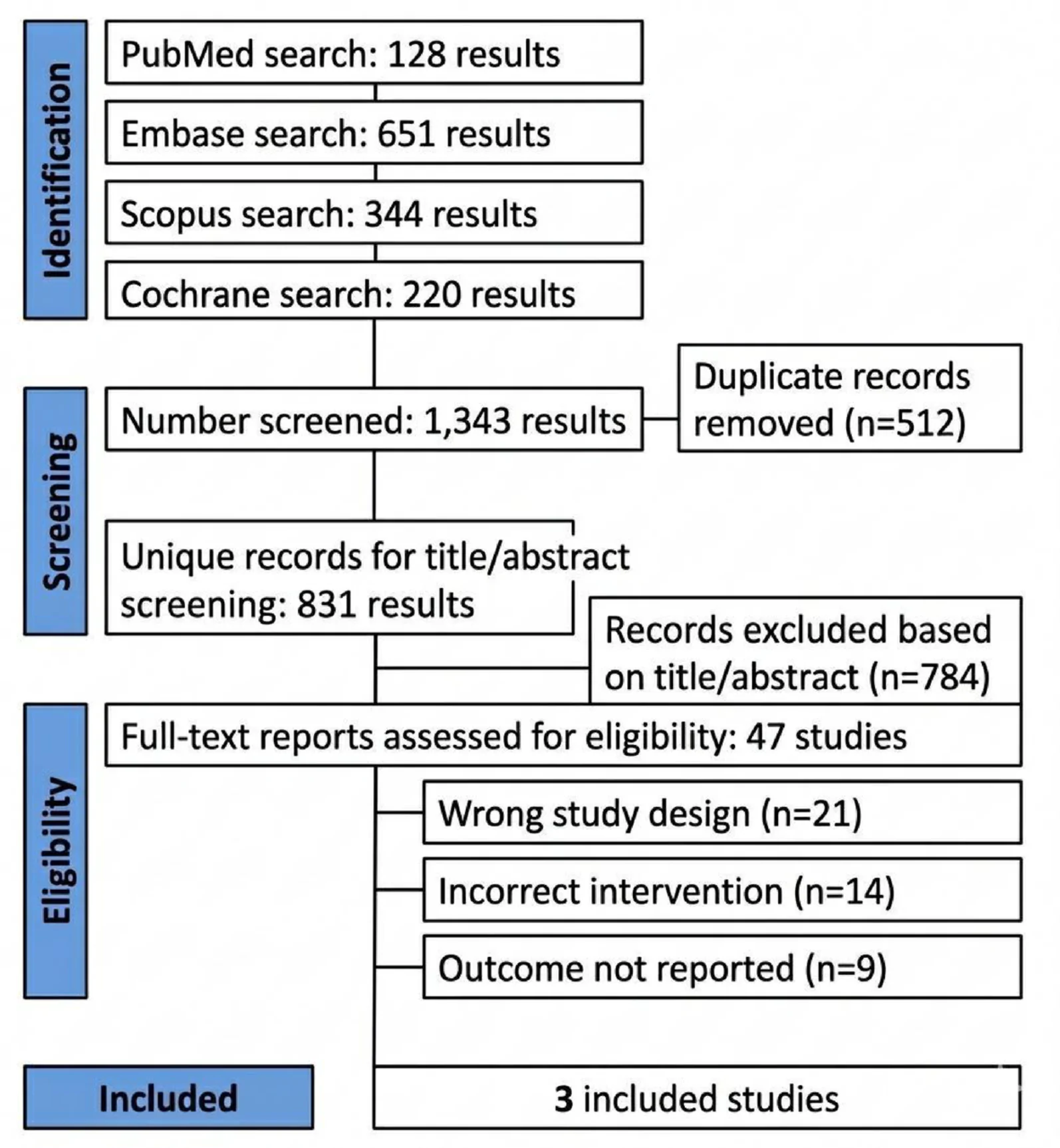
PRISMA 2020 flow diagram of the study selection process

A systematic literature search was conducted across PubMed, Embase, Scopus, and the Cochrane Library, yielding a total of 1,343 records. After removal of 512 duplicates, 831 unique records were screened based on titles and abstracts. Of these, 784 studies were excluded for not meeting the predefined inclusion criteria. The remaining 47 full-text articles underwent detailed eligibility assessment, resulting in the exclusion of 44 studies due to wrong study design (*n* = 21), incorrect intervention (*n* = 14), or outcome not reported (*n* = 9). Ultimately, three studies satisfied all eligibility criteria and were included in the qualitative and quantitative synthesis (Fig. 1).

### Baseline characteristics

Across the included randomized trials, baseline characteristics were generally well balanced between the mazdutide and dulaglutide groups. The total sample size varied across studies, with comparable distribution across treatment arms within each trial. The mean age of participants ranged approximately from 50 to 52 years, with a slight predominance of male participants (approximately 50–60%) across all groups. Baseline glycemic parameters were similar between groups, with mean HbA1c values consistently around 8.1% to 8.4%, and fasting plasma glucose levels approximately 8.5 to 9.5 mmol/L. Anthropometric measures were also comparable, with mean body weight ranging from approximately 75 to 85 kg and body mass index (BMI) between 27 and 30 kg/m² across studies. Blood pressure profiles were balanced, with mean systolic blood pressure around 120–130 mmHg and diastolic blood pressure approximately 75–85 mmHg. The mean duration of diabetes was similar across treatment arms, generally ranging from 4 to 6 years. Additionally, the proportion of patients receiving background metformin therapy was comparable between groups, typically exceeding 70% in most cohorts. Background antidiabetic therapy differed slightly across the three trials. Jiang et al. and Zhang et al. restricted background therapy to metformin alone (or diet and exercise), whereas Guo et al. additionally permitted metformin combined with an SGLT2 inhibitor or sulfonylurea, used by approximately 26% of participants in that trial. Overall, the consistency of baseline demographic and clinical characteristics across treatment groups suggests adequate comparability and minimizes the likelihood of confounding in the pooled efficacy and safety analyses (Table 1).

**Table 1:** Characteristics of included studies

Characteristic	Zhang 2024 MZD 3 mg [14]	Zhang 2024 MZD 4.5 mg [14]	Zhang 2024 MZD 6 mg [14]	Zhang 2024 Dulaglutide 1.5 mg	Jiang 2022 MZD 3 mg [43]	Jiang 2022 MZD 4.5 mg [43]	Jiang 2022 MZD 6 mg [43]	Jiang 2022 Dulaglutide	Guo 2025 MZD 4 mg [15]	Guo 2025 MZD 6 mg [15]	Guo 2025 Dulaglutide 1.5 mg [15]
**n**	51	49	49	50	8	8	8	6	242	244	245
**Age (years)**	52.5 (13.4)	55.1 (10.7)	54.4 (9.8)	52.8 (12.7)	58.6 (5.3)	47.9 (8.9)	54.6 (10.6)	50.7 (4.8)	51.7 (11.5)	52.1 (11.6)	51.7 (10.5)
**Male sex**,** n (%)**	26 (51.0)	32 (65.3)	36 (73.5)	26 (52.0)	6 (75.0)	3 (37.5)	5 (62.5)	6 (100)	155 (64.0)	150 (61.5)	137 (55.9)
**Female sex**,** n (%)**	25 (49.0)	17 (34.7)	13 (26.5)	24 (48.0)	2 (25.0)	5 (62.5)	3 (37.5)	0 (0)	87 (36.0)	94 (38.5)	108 (44.1)
**HbA1c (%)**	8.11 (0.84)	8.11 (0.91)	7.94 (0.93)	7.94 (0.92)	8.9 (0.7)	8.8 (1.0)	8.5 (1.1)	8.3 (1.4)	8.21 (0.83)	8.21 (0.86)	8.23 (0.86)
**Fasting plasma glucose (mmol/L)**	9.3 (2.5)	9.3 (1.9)	9.6 (2.2)	8.7 (2.0)	11.1 (1.9)	10.7 (1.8)	11.7 (2.2)	11.2 (3.1)	9.35 (2.29)	9.17 (2.05)	9.19 (2.14)
**Body weight (kg)**	78.1 (18.7)	75.3 (13.8)	76.3 (13.2)	72.3 (12.8)	65.5 (9.2)	70.9 (14.5)	69.9 (9.7)	75.1 (10.2)	76.0 (13.2)	78.1 (16.2)	76.8 (15.2)
**BMI (kg/m²)**	28.0 (4.8)	27.3 (3.8)	27.2 (3.4)	26.7 (3.2)	24.1 (1.4)	25.7 (3.4)	26.0 (2.2)	26.6 (3.0)	27.6 (3.6)	28.1 (4.1)	28.0 (4.1)
**Systolic BP (mmHg)**	128.5 (13.1)	130.5 (11.2)	128.9 (11.7)	125.6 (13.3)	129.9 (14.3)	120.8 (11.0)	122.1 (15.9)	115.8 (10.1)	125.2 (12.4)	126.2 (11.8)	125.1 (12.4)
**Diastolic BP (mmHg)**	82.3 (8.6)	82.6 (7.9)	82.6 (8.1)	80.9 (8.6)	82.3 (12.2)	81.9 (7.6)	75.3 (9.0)	76.3 (7.8)	81.7 (7.1)	82.4 (7.0)	83.0 (7.5)
**Diabetes duration (years)**	3.80(2.96)	4.57(3.93)	3.37 (2.96)	4.07(2.96)	1.93 (0.96)	4.13(2.44)	6.07 (5.04)	2.77(1.33)	5.1 (4.4)	5.7 (4.7)	5.3 (4.5)
**Metformin use**,** n (%)**	35 (68.6)	33 (67.3)	32 (65.3)	33 (66.0)	3 (37.5)	4 (50.0)	4 (50.0)	4 (66.7)	178 (73.6)	181 (74.2)	180 (73.5)

### Efficacy outcomes

#### Change from baseline (%) in HbA1c

**Fig. 2:**
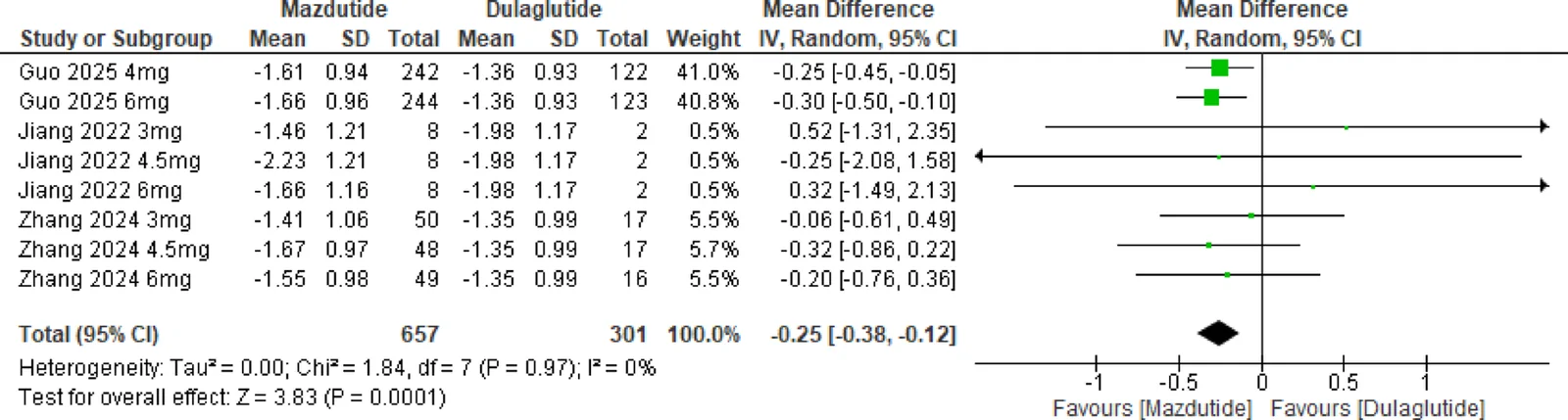
Change from baseline (%) in HbA1c

The pooled analysis demonstrated that mazdutide was associated with a significantly greater reduction in HbA1c baseline percentage change compared with dulaglutide (Mean Difference [MD]: -0.25; 95% CI: -0.38 to -0.12; *p* = 0.0001) (Fig. 2) **(**Table 2**).** There was no evidence of statistical heterogeneity among the included studies (I² = 0%), indicating consistent effects across trials. This indicates that mazdutide provides modestly but reliably better glycemic control than dulaglutide over the studied follow-up period.

**Table 2:**
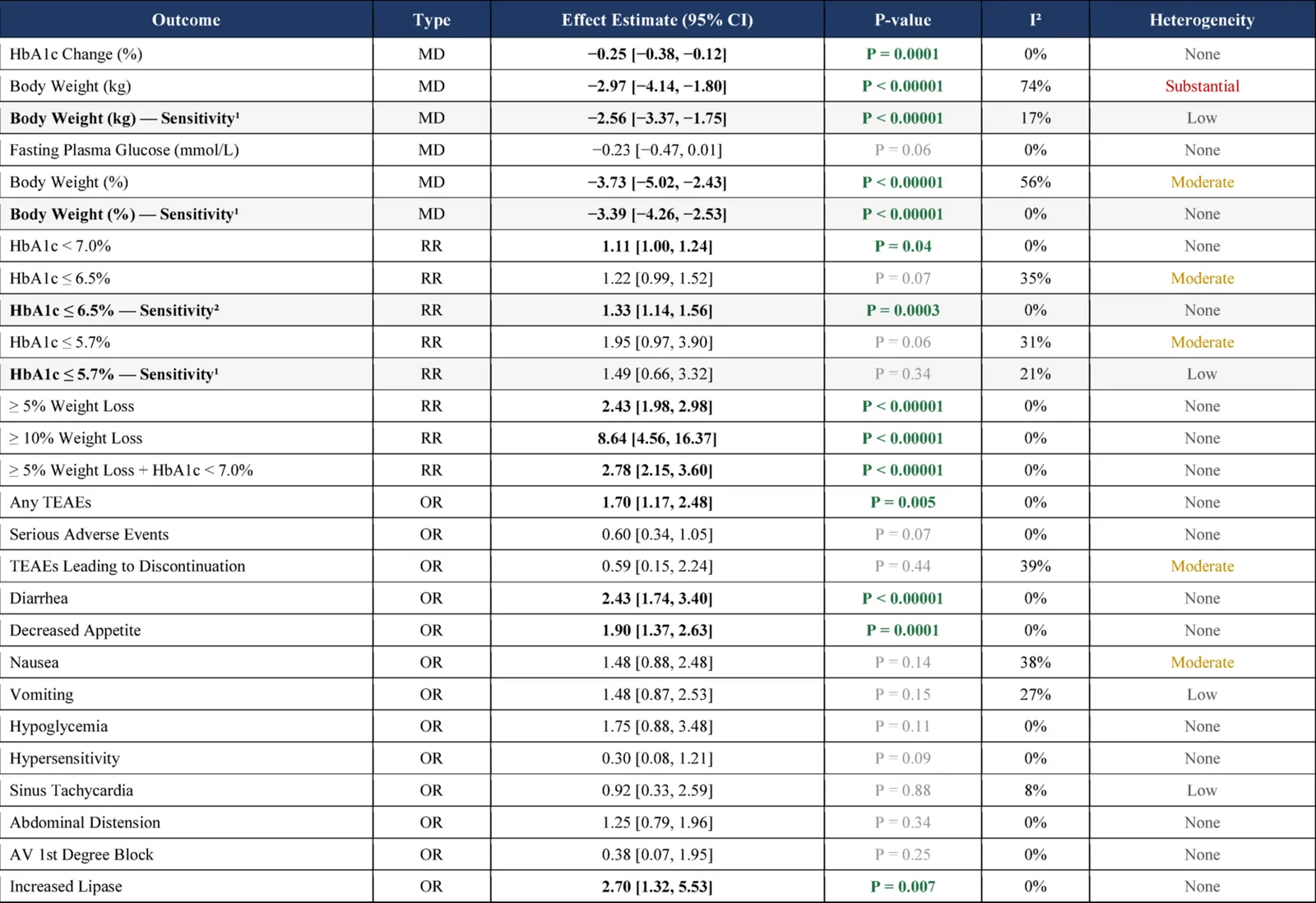
Summary of pooled efficacy and safety outcomes for mazdutide versus dulaglutide

¹ Sensitivity analysis with Guo 2025 6 mg removed. ² Sensitivity analysis with Zhang 2024 3 mg removed

Bold P-values (green) indicate statistically significant overall effect (*P* < 0.05). Shaded rows indicate sensitivity-analysis variants of the row immediately above them

**Fig. 3:**
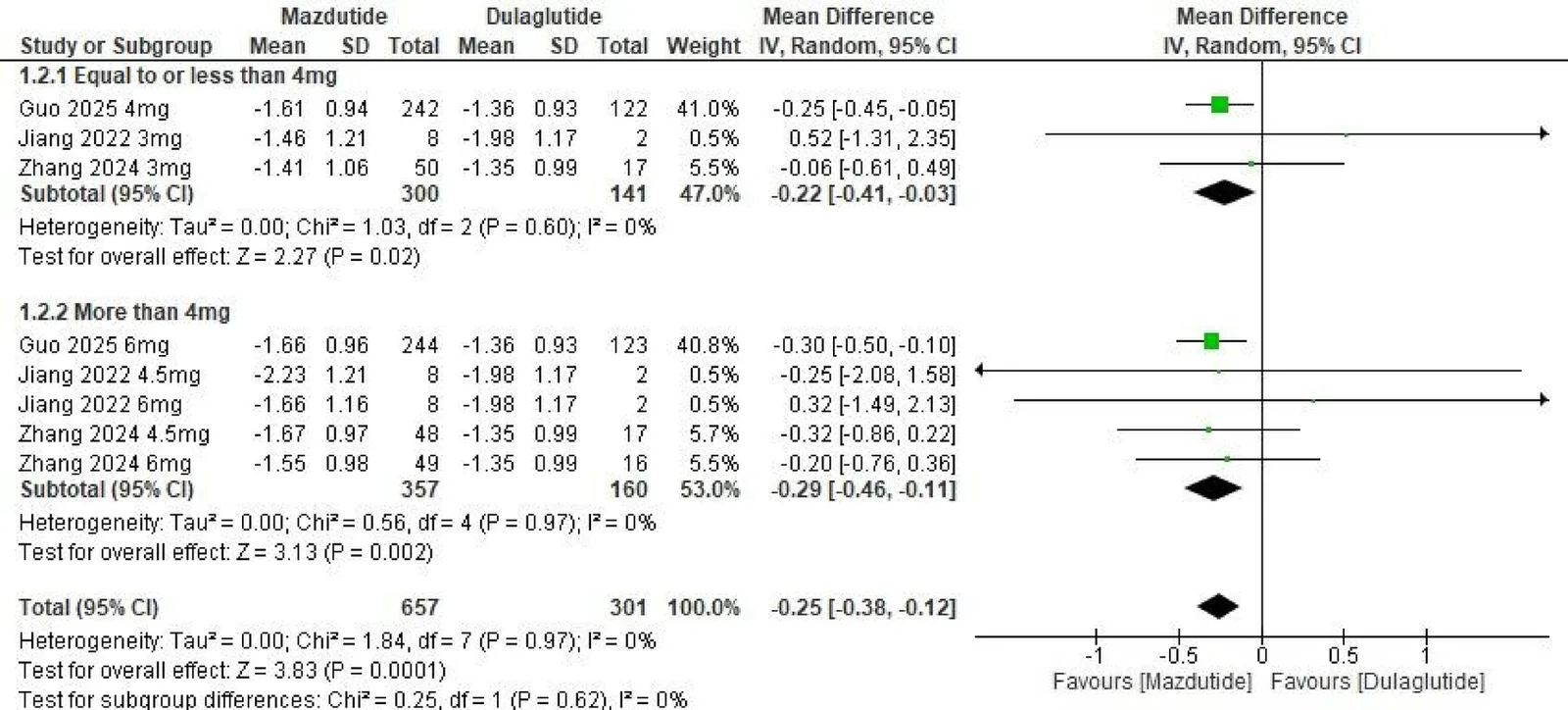
Change from baseline (%) in HbA1c (Subgrouped by Dosage: ≤ 4 mg and > 4 mg)

The subgroup analysis on the basis of mazdutide dosing did not reach statistical significance (*p* = 0.62) (Fig. 3). Similarly, subgroup analysis stratified by treatment duration did not demonstrate a significant difference between trials lasting ≤ 20 weeks and those lasting > 20 weeks (*p* = 0.51) (All the additional outcomes and subgroup data are summarized in (Supplementary Table 2).

#### Change from baseline (kg) in body weight

**Fig. 4:**
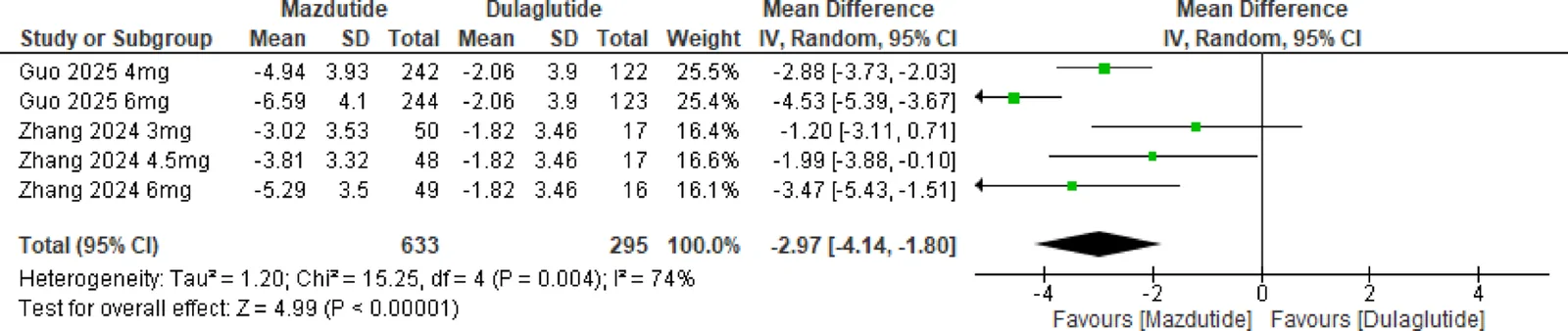
Change from baseline (Kg) in body weight

**Fig. 5:**
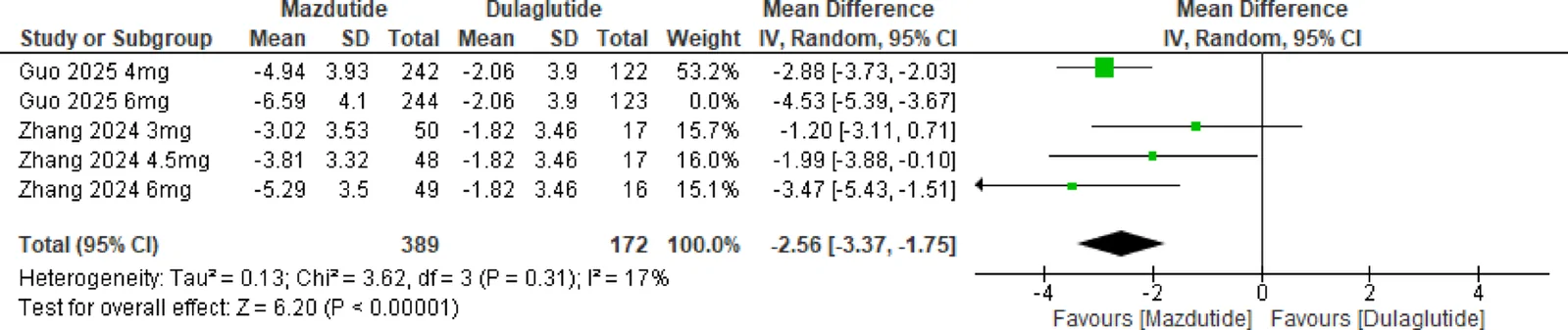
Change from baseline (Kg) in body weight with Sensitivity Analysis (When Guo 2025 6 mg is removed)

Mazdutide demonstrates a statistically significant and clinically meaningful reduction in body weight compared to Dulaglutide, with a mean difference of -2.97 kg (95% CI: -4.14 to -1.80; *p* < 0.00001, I² = 74%) (Fig. 4). Sensitivity analysis, excluding the Guo 2025 6 mg study, yielded a revised mean difference of -2.56 (95% CI: -3.37 to -1.75; *p* < 0.00001) (Fig. 5). These findings confirm the superior efficacy of mazdutide over dulaglutide in reducing baseline body weight, with reduced heterogeneity (I² = 17%).

Subgroup analysis did not show a meaningful difference across varying mazdutide doses (*p* = 0.27). When stratified by treatment duration, the subgroup differences were not statistically significant (*p* = 0.16), although a trend toward greater absolute weight reduction was noted in trials lasting > 20 weeks (MD: -3.70 kg) compared to those ≤ 20 weeks (MD: -2.20 kg).

#### Change from baseline (mmol·L⁻¹) in fasting plasma glucose

**Fig. 6:**
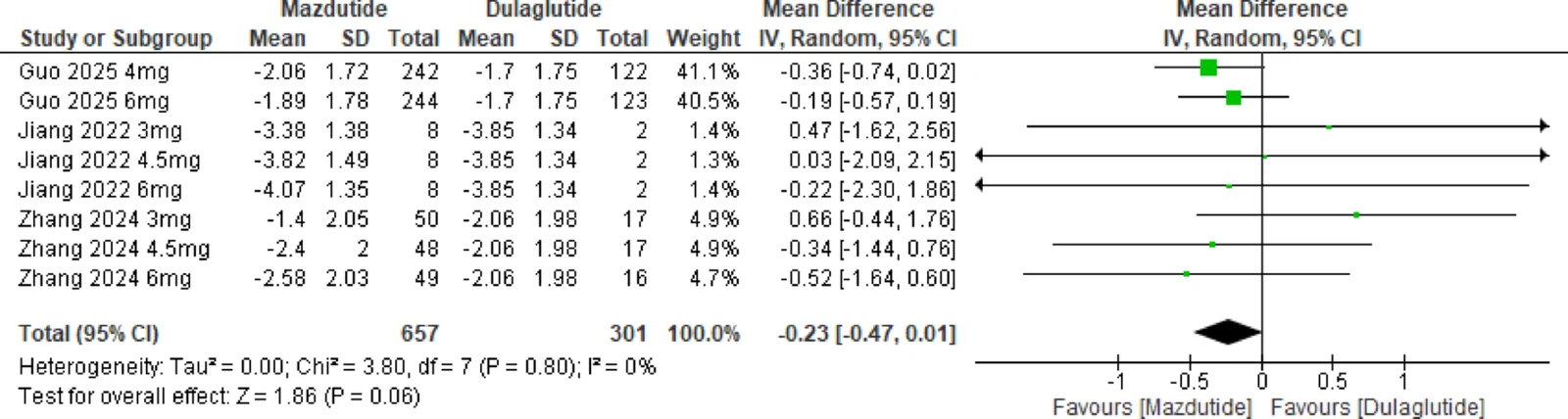
Change from baseline (mmol.l^−1^) in Fasting Plasma Glucose

The pooled results did not demonstrate a statistically significant greater reduction in fasting plasma glucose with mazdutide compared with dulaglutide (MD: -0.23 mmol·L⁻¹; 95% CI: -0.47 to 0.01; *p* = 0.06) (Fig. 6). Heterogeneity across studies was low (I² = 0%) indicating consistent effects across trials. When analyzed by mazdutide dosage, the subgroup differences were not statistically significant (*p* = 0.56). Subgroup analysis stratified by treatment duration also failed to demonstrate a statistically significant difference between the subgroups (*p* = 0.44).

#### Participants achieving HbA1c < 7.0%

In pooled analyses, patients receiving mazdutide were significantly more likely to attain HbA1c < 7.0% than those treated with dulaglutide (RR: 1.11; 95% CI: 1.00 to 1.24; *p* = 0.04). The absence of heterogeneity among studies (I² = 0%) indicates that this effect was consistent across trials. This demonstrates the superior glycemic efficacy of mazdutide over dulaglutide during the observed period.

The subgroup analysis stratified by mazdutide dose did not demonstrate statistical significance (*p* = 0.60). Furthermore, subgroup evaluation based on treatment duration showed no statistically significant difference between the time-point groups (*p* = 0.79).

#### Participants achieving HbA1c ≤ 6.5%

The pooled analysis demonstrated a numerically higher likelihood of achieving HbA1c ≤ 6.5% with mazdutide compared with dulaglutide, although this difference was not statistically significant (RR: 1.22; 95% CI: 0.99 to 1.52; *p* = 0.07, I² = 35%). Sensitivity analysis, excluding Zhang 2024 (3 mg), yielded a statistically significant effect (RR: 1.33; 95% CI: 1.14 to 1.56; *p* = 0.0003) with no observed heterogeneity (I² = 0%), indicating consistent results across studies.

The effect of mazdutide dose in subgroup analysis was not statistically significant (*p* = 0.31). Similarly, subgroup analysis by treatment duration did not demonstrate a statistically significant difference between time points (*p* = 0.15).

#### Participants achieving HbA1c ≤ 5.7%

The pooled estimate suggested a higher likelihood of achieving HbA1c ≤ 5.7% with mazdutide compared with dulaglutide; however, this did not reach statistical significance (RR: 1.95; 95% CI: 0.97 to 3.90; *p* = 0.06) and was accompanied by moderate heterogeneity (I² = 31%). Sensitivity analysis excluding Guo 2025 (6 mg) demonstrated a non-significant effect (RR: 1.49; 95% CI: 0.66 to 3.32; *p* = 0.34) with decreased but still substantial heterogeneity (I² = 21%).

Subgroup analysis according to mazdutide dose failed to show a significant effect (*p* = 0.89). In contrast, subgrouping by treatment duration revealed a statistically significant subgroup difference (*p* = 0.02), driven entirely by a strong, significant effect in trials extending beyond 20 weeks (RR: 3.36; 95% CI: 1.76 to 6.42; *p* = 0.0002) compared to early time points (≤ 20 weeks: RR: 0.82; 95% CI: 0.30 to 2.20; *p* = 0.69).

#### Participants achieving ≥ 5% weight loss

Mazdutide treatment was associated with a significantly higher probability of attaining ≥ 5% weight loss than dulaglutide (RR: 2.43; 95% CI: 1.98 to 2.98; *p* < 0.00001). Between-study variability was minimal (I² = 0%), indicating consistent results across trials. Overall, mazdutide effectively enhances the likelihood of achieving clinically relevant weight reduction.

No statistically significant difference was observed in the subgroup analysis based on mazdutide dosing (*p* = 0.19). Similarly, the subgroup analysis based on treatment duration was not statistically significant (*p* = 0.65), showing powerful and consistent benefits at both ≤ 20 weeks (RR: 2.11; 95% CI: 1.13 to 3.96) and > 20 weeks (RR: 2.47; 95% CI: 1.92 to 3.17).

#### Participants achieving ≥ 10% weight loss

Compared with dulaglutide, mazdutide significantly improved the likelihood of participants achieving ≥ 10% weight loss (RR: 8.64; 95% CI: 4.56 to 16.37; *p* < 0.00001). Between-study variability was negligible (I² = 0%), suggesting reproducible effects across the included trials. These results underscore the enhanced effectiveness of mazdutide in promoting clinically meaningful weight loss.

Subgroup analysis according to mazdutide dose failed to show a significant effect (*p* = 0.61). Subgroup analysis stratified by treatment duration also did not show a statistically significant difference between groups (*p* = 0.59), confirming robust relative benefits regardless of follow-up length.

#### Participants achieving ≥ 5% weight loss and HbA1c < 7.0%

Mazdutide significantly increased the probability of achieving ≥ 5% weight loss and HbA1c < 7.0% relative to dulaglutide (RR: 2.78; 95% CI: 2.15 to 3.60; *p* < 0.00001). Heterogeneity among studies was minimal (I² = 0%), reflecting consistent outcomes across trials. Overall, these results support the dual efficacy of mazdutide in promoting weight loss and glycemic improvement.

The subgroup analysis by mazdutide dosage did not reveal a statistically significant difference (*p* = 0.27). Furthermore, stratification by treatment duration demonstrated that subgroup differences were not statistically significant (*p* = 0.73), verifying a consistent composite benefit across time points.

#### Change from baseline (%) in body weight

The pooled analysis demonstrates that mazdutide was associated with a significantly greater reduction in percentage body weight compared with dulaglutide (MD: -3.73; 95% CI: -5.02 to -2.43; *p* < 0.00001). High statistical heterogeneity was observed among the included studies (I² = 56%), indicating substantial variability in treatment effects across the trials. These findings confirm the superior efficacy of mazdutide over dulaglutide in achieving percentage body weight reduction. Sensitivity analysis, excluding the Guo 2025 6 mg study, yielded a revised mean difference of -3.39 (95% CI: -4.26 to -2.53; *p* < 0.00001). This exclusion significantly reduced the statistical heterogeneity (I² = 0%), suggesting that the Guo 2025 6 mg trial was a primary contributor to the initial inconsistency.

Subgroup analysis based on mazdutide dosing did not reach statistical significance (*p* = 0.09). When stratified by treatment duration, the subgroup differences were not statistically significant (*p* = 0.11), with a greater point estimate of reduction at longer durations (> 20 weeks: MD: -4.77%) compared to shorter ones (≤ 20 weeks: MD: -2.82%).

### Safety outcomes

#### Any treatment emergent adverse events (TEAEs)

Mazdutide is associated with a statistically significant higher risk of treatment emergent adverse events compared to Dulaglutide, with an odds ratio of 1.70 (95% CI: 1.17 to 2.48; *p* = 0.005). No significant heterogeneity was observed between the studies (I² = 0%), indicating consistent results across the included trials. These findings suggest that while mazdutide may offer clinical benefits, it is associated with an increased risk of treatment emergent adverse events.

No significant between-group differences were observed upon stratification by mazdutide dose (*p* = 0.66). Similarly, subgroup analysis by treatment duration did not reveal any significant differences between the time-point groups (*p* = 0.59) (Supplementary Table 3).

#### Serious adverse events (SAEs)

Mazdutide shows a trend towards a lower risk of serious adverse events compared to Dulaglutide, with an odds ratio of 0.60 (95% CI: 0.34 to 1.05; *p* = 0.07). Although the overall effect did not reach statistical significance, no heterogeneity was observed between the studies (I² = 0%), suggesting consistent findings across the trials. These results indicate that mazdutide may be associated with a lower risk of serious adverse events, although further studies are required to confirm this finding.

Variation in mazdutide dose was not associated with statistically significant differences in subgroup outcomes (*p* = 0.55). Subgroup analysis according to treatment duration also failed to show a significant difference between groups (*p* = 0.39).

#### Teaes leading to treatment discontinuation

Mazdutide is associated with a lower likelihood of treatment discontinuation due to treatment-emergent adverse events (TEAEs) compared to Dulaglutide, with an odds ratio of 0.59 (95% CI: 0.15 to 2.24; *p* = 0.44). The result did not reach statistical significance, suggesting that there is no conclusive evidence to indicate a substantial difference between the two treatments in terms of discontinuation rates due to TEAEs. There was low heterogeneity observed across the studies (I² = 39%), indicating consistent findings.

Subgroup findings across mazdutide doses were statistically non-significant (*p* = 0.89). Additionally, stratification by treatment duration reveals a statistically significant difference between subgroups (*p* = 0.01).

#### Diarrhea

Mazdutide is associated with a significantly increased likelihood of diarrhea among patients compared to dulaglutide, with an odds ratio of 2.43 (95% CI: 1.74 to 3.40; *p* < 0.00001). This result demonstrates statistical significance, indicating a clear relationship between mazdutide use and an elevated risk of diarrhea. The absence of heterogeneity (I² = 0%) across the studies suggests that these findings are consistent and robust. These findings underscore that, although mazdutide may offer therapeutic benefits, it is linked to a higher incidence of diarrhea relative to dulaglutide.

Dose-based subgroup comparisons did not demonstrate a significant association (*p* = 0.67). Subgroup analysis by treatment duration also did not show a statistically significant difference between the time points (*p* = 0.82).

#### Decreased appetite

Mazdutide is significantly more likely to cause decreased appetite compared to Dulaglutide, with an odds ratio of 1.90 (95% CI: 1.37 to 2.63; *p* = 0.0001). The result is statistically significant, indicating a strong association between Mazdutide and an increased risk of decreased appetite. There is no heterogeneity across the studies (I² = 0%), suggesting consistent findings. These results imply that mazdutide is associated with a higher incidence of decreased appetite compared to dulaglutide.

Mazdutide dose stratification did not significantly influence the decreased appetite outcomes (*p* = 0.69). Stratification by treatment duration confirmed that the subgroup differences were not statistically significant (*p* = 0.72).

#### Nausea

Mazdutide is associated with a slightly higher chance of nausea compared to dulaglutide, with an odds ratio of 1.48 (95% CI: 0.88 to 2.48; *p* = 0.14), though the difference was not statistically significant. The heterogeneity between the studies was moderate (I² = 38%), suggesting some inconsistency in the findings. Although Mazdutide could be linked to a greater risk of nausea, the evidence remains insufficient to make a conclusive determination.

Dose-based subgroup comparisons did not demonstrate a significant association (*p* = 0.78). When analyzed by treatment duration, the subgroup difference was statistically significant (*p* = 0.001), with a numerically higher odds ratio was noted in trials > 20 weeks (OR: 2.60; 95% CI: 1.78 to 3.81).

#### Vomiting

There is no significant association between mazdutide and a higher risk of vomiting compared to Dulaglutide, with an odds ratio of 1.48 (95% CI: 0.87 to 2.53; *p* = 0.15). The heterogeneity between studies was low (I² = 27%). Analysis stratified by mazdutide dosing showed no statistically significant variation (*p* = 0.78). Subgroup analysis on the basis of treatment duration demonstrates a statistically significant difference between the subgroups (*p* = 0.02).

#### Hypoglycemia

Mazdutide showed higher but not statistically significant odds of hypoglycemia compared to dulaglutide (OR: 1.75; 95% CI: 0.88–3.48; *p* = 0.11). No statistical heterogeneity was observed across studies (I² = 0%), However, hypoglycemia definitions and severity grading varied across the included trials, and this heterogeneity in case ascertainment should be considered when interpreting the pooled estimate. Overall, these results do not demonstrate a statistically significant difference between treatments, although a higher point estimate was observed with mazdutide.

No statistically significant subgroup effect was detected when stratified by mazdutide dose (*p* = 0.84). Subgroup differences by treatment duration were also not statistically significant (*p* = 0.57).

#### Hypersensitivity

Mazdutide is associated with a reduced likelihood of hypersensitivity reactions compared to dulaglutide, with an odds ratio of 0.30 (95% CI: 0.08 to 1.21; *p* = 0.09). Although the result did not achieve statistical significance, there was no heterogeneity observed between studies (I² = 0%), suggesting consistency in the findings across trials. Overall results suggest potentially a lower risk of hypersensitivity with mazdutide, but the result is not statistically significant.

The subgroup analysis failed to identify significant differences across mazdutide dosing levels (*p* = 0.58). Subgroup analysis across treatment duration groups was also statistically non-significant (*p* = 0.11).

#### Sinus tachycardia

There is a marginally decreased likelihood of sinus tachycardia with mazdutide compared to dulaglutide, with an odds ratio of 0.92 (95% CI: 0.33 to 2.59; *p* = 0.88). Although this result did not achieve statistical significance, minimal heterogeneity was observed across studies (I² = 8%), indicating consistency in the findings. The results suggest a potential lower risk of sinus tachycardia with mazdutide, though this remains statistically insignificant.

No significant between-group differences were observed upon stratification by mazdutide dose (*p* = 0.20). Furthermore, the subgroup analysis based on treatment duration did not reach statistical significance (*p* = 0.14).

#### Abdominal distension

Mazdutide may be linked to a slightly increased risk of abdominal distension compared to dulaglutide, with an odds ratio of 1.25 (95% CI: 0.79 to 1.96; *p* = 0.34). Although the result did not reach statistical significance, the studies showed no heterogeneity (I² = 0%), which suggests consistency in the data. This points to a potentially higher risk of abdominal distension with mazdutide, but this remains statistically insignificant.

Dose-based subgroup comparisons did not demonstrate a significant association (*p* = 0.60). Similarly, subgroup differences based on treatment duration were not statistically significant (*p* = 0.48).

#### AV 1st degree block

Mazdutide may be associated with a modestly reduced risk of 1st degree AV block compared to dulaglutide, as indicated by an odds ratio of 0.38 (95% CI: 0.07 to 1.95; *p* = 0.25). While this result did not achieve statistical significance, the absence of heterogeneity across the studies (I² = 0%) suggests that the findings are consistent. These results point to a potential lower risk of 1st degree AV block with mazdutide; however, this difference remains statistically insignificant.

Subgroup findings across mazdutide doses were statistically non-significant (*p* = 0.38). Note: A subgroup analysis based on treatment duration was not possible for this outcome due to data constraints.

#### Lipase levels

Compared to dulaglutide, mazdutide is potentially associated with a higher risk of elevated lipase levels, with an odds ratio of 2.70 (95% CI: 1.32 to 5.53; *p* = 0.007). The absence of heterogeneity (I² = 0%) across the studies indicates that the findings are consistent, supporting a potential increase in lipase levels with mazdutide.

No significant between-group differences were observed upon stratification by mazdutide dose (*p* = 0.49). Subgroup analysis according to treatment duration also failed to show a statistically significant difference between the time points (*p* = 0.84).

### Certainty of evidence

The certainty of evidence across outcomes, assessed using the GRADE framework, ranged from low to high. High-certainty evidence supported the superiority of GLP-1 receptor agonists over comparators for glycaemic outcomes (HbA1c reduction, achievement of HbA1c < 7.0%), clinically meaningful weight loss thresholds (≥ 5% and ≥ 10% body weight loss), the composite endpoint of ≥ 5% weight loss with HbA1c < 7.0%, and selected safety signals including any treatment-emergent adverse events (TEAEs), diarrhoea, decreased appetite, and increased lipase. Moderate-certainty evidence was observed for fasting plasma glucose, HbA1c ≤ 6.5%, HbA1c ≤ 5.7%, serious adverse events, nausea, vomiting, hypoglycaemia and abdominal distension, primarily owing to 95% confidence intervals that crossed the line of no effect or approached clinically important thresholds (imprecision). Evidence for body weight reduction in absolute (kg) and percentage terms was downgraded to low certainty due to very serious inconsistency, reflecting substantial between-trial heterogeneity (I² of 74% and 56%, respectively), suggesting variability in the magnitude of weight effect across included trials. Similarly, TEAEs leading to treatment discontinuation, Sinus Tachycardia, hypersensitivity reactions, and AV first-degree block were rated as low certainty due to very serious imprecision attributable to sparse events, wide confidence intervals, and statistically unstable estimates. No concerns regarding risk of bias, indirectness, or publication bias were identified across any of the assessed outcomes (Supplementary Table 4).

### Risk of bias assessment

**Fig. 7:**
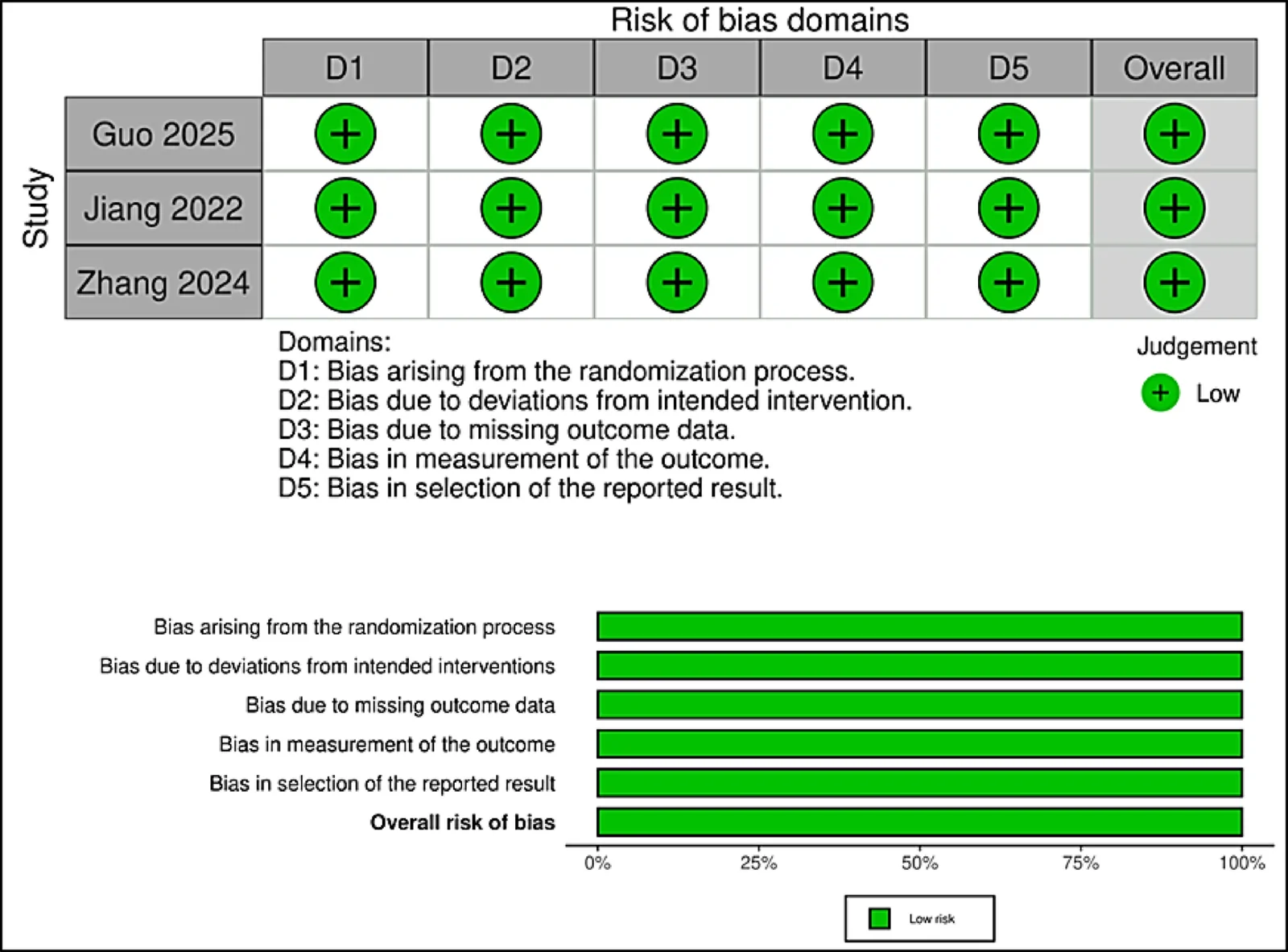
Summary of risk of bias assessment for efficacy outcomes

The methodological quality of the included randomized controlled trials was evaluated using the Cochrane Risk of Bias (RoB 2) tool across five domains. For efficacy outcomes, all included studies (Guo 2025, Jiang 2022, and Zhang 2024) were judged to have a low risk of bias across all domains, including randomization, deviations from intended interventions, missing outcome data, outcome measurement, and selective reporting. The summary assessment demonstrated that 100% of studies were rated as low risk, reflecting high methodological rigor, absence of selective reporting, and consistency in study conduct. This supports a high level of confidence in the pooled efficacy estimates (Fig. 7).

In contrast, for safety outcomes, while all studies maintained a low risk of bias in domains related to randomization (D1), deviations from interventions (D2), missing data (D3), and selective reporting (D5), some concerns were identified in the measurement of outcomes domain (D4) across all trials. This was primarily attributable to potential limitations in outcome ascertainment or lack of blinding for adverse event reporting. Consequently, the overall risk of bias for safety outcomes was judged as “some concerns” for all included studies, although no domain was rated as high risk (Supplementary Fig. 1).

Overall, these findings indicate high certainty of evidence for efficacy outcomes and moderate certainty for safety outcomes, with the latter primarily limited by consistent concerns related to outcome measurement. Despite this, the absence of high-risk bias domains and the consistency of findings across studies support the overall robustness and reliability of the pooled analyses.

## Discussion

In this meta-analysis of RCTs comparing mazdutide with dulaglutide in adults with type 2 diabetes mellitus, mazdutide demonstrated superior overall metabolic efficacy, with the clearest advantage seen in body weight-related outcomes. It produced greater reductions in HbA1c and body weight, along with a numerical but non-significant reduction in fasting plasma glucose, and significantly increased the likelihood of achieving HbA1c < 7.0%, ≥ 5% weight loss, ≥ 10% weight loss, and the composite endpoint of ≥ 5% weight loss with HbA1c < 7.0%. Although HbA1c ≤ 6.5% and HbA1c ≤ 5.7% were not consistently significant in the primary analyses, the overall direction generally favored mazdutide, and HbA1c ≤ 6.5% became significant after sensitivity analysis. On the safety side, mazdutide was associated with significantly higher rates of treatment-emergent adverse events, diarrhea, decreased appetite, and lipase elevation, whereas serious adverse events, discontinuation due to adverse events, nausea, vomiting, hypoglycemia, hypersensitivity, sinus tachycardia, abdominal distension, and first-degree atrioventricular block did not differ significantly from dulaglutide. As only three trials, all from a single sponsor and a single national population, met inclusion criteria, these findings should be regarded as preliminary, and the discussion below is framed with this limited evidence base in mind.

### Efficacy outcomes

The efficacy pattern observed in our meta-analysis is biologically plausible and consistent with the pharmacologic rationale of GLP-1/glucagon-based co-agonism [22–24]. GLP-1 receptor agonism improves glycemic control through glucose-dependent insulin secretion, suppression of glucagon during hyperglycemia, delayed gastric emptying, and reduced caloric intake, whereas glucagon receptor agonism may contribute to greater energy expenditure, altered fat metabolism, and weight reduction [22, 23]. Reviews of glucagon-based co-agonists have suggested that their main therapeutic promise lies in producing substantial weight loss and broader metabolic effects while maintaining glycaemic benefit [23, 24]. Our pooled findings fit that framework well, as the glycemic advantage of mazdutide over dulaglutide was modest but consistent, whereas the separation in weight-related outcomes was substantially greater.

Mazdutide significantly improved HbA1c change from baseline and increased the probability of achieving HbA1c < 7.0%, supporting a glycemic advantage over dulaglutide, although this conclusion rests on only three trials and warrants confirmation. However, the magnitude of that advantage appeared moderate rather than dramatic, which is clinically unsurprising given that dulaglutide is itself an effective once-weekly GLP-1 receptor agonist with established glucose-lowering efficacy [25, 26]. In active-comparator incretin trials, differences in glycemic outcomes are often narrower than differences in body weight [27, 28]. Our findings are consistent with that broader pattern.

This interpretation also helps explain the less stable findings for stricter glycemic thresholds. HbA1c ≤ 6.5% showed only a numerical benefit in the primary analysis, although it became significant after sensitivity analysis, while HbA1c ≤ 5.7% remained unstable and directionally inconsistent after exclusion of influential data. These results suggest that mazdutide probably improves the likelihood of reaching standard glycemic targets, but the current evidence is not yet strong enough to claim a robust advantage for near-normal HbA1c thresholds.Because responder outcomes based on strict HbA1c thresholds are influenced by baseline glycaemic status and diabetes duration some instability is not unexpected in a small evidence base [29, 30].

Mazdutide produced a numerically greater but statistically non-significant reduction in fasting plasma glucose, although the magnitude of the difference was modest. This complements the HbA1c findings and supports an overall glucose-lowering trend over dulaglutide without suggesting a dramatic fasting glucose effect. Such a pattern is broadly consistent with the known profile of incretin-based therapies, in which fasting glucose reductions are generally aligned with HbA1c changes [29], although the non-significant result in our analysis means this should be regarded as a supportive trend rather than a confirmed finding.

The most convincing efficacy signal in our review was body weight reduction. Mazdutide significantly reduced body weight and markedly increased the likelihood of achieving both ≥ 5% and ≥ 10% weight loss, with the ≥ 10% outcome showing a particularly large effect. This is consistent with the emerging literature on glucagon-based co-agonists, in which added non-GLP-1 receptor activity may yield a more pronounced effect on body weight than on incremental glycemic improvement [23, 31]. Similar patterns have been seen with other multi-agonist agents, where comparator separation is often more pronounced for body weight than for glycaemic outcomes [27, 31, 32].

The superiority of mazdutide for the composite endpoint of ≥ 5% weight loss and HbA1c < 7.0% is especially relevant clinically because it captures two important treatment goals in type 2 diabetes: glycaemic control and meaningful weight reduction [22, 30]. This suggests that the benefit of mazdutide is not limited to isolated laboratory improvement, but extends to a broader metabolic profile, including weight-related benefit, that may be more clinically meaningful than HbA1c alone [22, 30].

Body-weight change initially showed substantial heterogeneity, but sensitivity analysis materially reduced heterogeneity without changing the direction of effect. This suggests that mazdutide’s benefit for weight reduction is consistent in direction across analyses, though the exact magnitude may vary according to dose, study design, or population characteristics, and confirmation in additional independent trials is needed before this can be considered a robust finding. The absence of significant subgroup effects by mazdutide dose across efficacy outcomes should therefore be interpreted cautiously. In small meta-analyses, subgroup analyses often have low power, and failure to detect a significant interaction should not be interpreted as evidence of dose equivalence [33, 34].

### Safety outcomes

The overall safety profile suggests that mazdutide’s main tradeoff may be a greater tolerability burden rather than greater serious toxicity. Mazdutide significantly increased the risk of treatment-emergent adverse events, but serious adverse events and treatment discontinuation due to adverse events were not significantly different from dulaglutide. This distinction is clinically relevant because it suggests that mazdutide causes more side effects overall, but current evidence, limited to three trials, does not demonstrate that it causes more serious harm than dulaglutide; this absence of a signal should not be mistaken for proof of equivalent serious-harm risk.

The excess in overall adverse events appears to be driven mainly by gastrointestinal intolerance, which is consistent with the well-established safety profile of incretin-based therapies [25, 26, 35, 36]. Published analyses of GLP-1 receptor agonists show that nausea, vomiting, and diarrhea are among the most frequent adverse events and often show a dose-dependent pattern [33]. Dulaglutide-specific reviews and pooled analyses report a similar GI tolerability pattern [25, 26, 37].

In our pooled analysis, diarrhea was clearly more common with mazdutide. This is consistent with both class-level GLP-1 receptor agonist data and dulaglutide-specific evidence, in which diarrhea is among the most frequently reported gastrointestinal adverse events [26, 27, 35, 37]. Because dulaglutide itself already causes diarrhea, the persistence of a significant difference in a head-to-head comparison is suggestive of a differentiating adverse effect of mazdutide rather than merely a background class effect, although confirmation in additional trials would strengthen this interpretation.

Decreased appetite was also significantly more frequent with mazdutide. From a safety perspective this is an adverse event, but mechanistically it likely reflects the same biology that underlies the drug’s superior weight-loss efficacy. Appetite suppression is commonly described as one of the pathways through which incretin-based and co-agonist therapies promote weight reduction [22, 23]. This finding is therefore consistent with the overall efficacy profile of mazdutide rather than contradictory to it.

Nausea did not differ significantly between mazdutide and dulaglutide, despite being a well-recognized adverse effect of GLP-1-based therapies [25, 26, 35, 37]. This should not be interpreted as evidence that nausea is absent with mazdutide; rather, dulaglutide is itself associated with nausea, and a head-to-head comparison may reduce the observable between-group difference [25, 26, 37]. The moderate heterogeneity seen for this outcome may reflect between-trial differences in study design and adverse-event reporting.

Vomiting, in contrast, was numerically but not significantly more common with mazdutide, and showed low-to-moderate heterogeneity, suggesting a directionally consistent but inconclusive difference from dulaglutide. This supports the interpretation that mazdutide’s gastrointestinal burden extends beyond appetite suppression or mild lower-GI symptoms and may include a modest increase in upper gastrointestinal intolerance, though current evidence does not establish this conclusively.

Abdominal distension was not significantly different between the two groups. Given that this event has also been reported with dulaglutide and other GLP-1 receptor agonists, the lack of a significant difference is plausible and suggests that abdominal distension may represent a background class-related gastrointestinal effect rather than a mazdutide-specific signal.

Hypoglycemia was numerically more common with mazdutide, although the difference did not reach statistical significance, and deserves cautious interpretation. GLP-1 receptor agonists generally have a low intrinsic risk of hypoglycemia because their insulinotropic effects are glucose-dependent [22, 26]. In prior studies, dulaglutide has also generally been associated with a low inherent risk of hypoglycemia [25, 26]. The observed non-significant signal in our analysis therefore should not be overinterpreted as proof of any hypoglycemia liability; it may partly reflect small event counts, differences in background oral therapies, or reporting thresholds. Nevertheless, because the point estimate favored a higher risk with mazdutide despite the lack of statistical significance, this finding warrants attention in future studies, particularly when mazdutide is used with agents that independently increase hypoglycemia risk.

Lipase elevation was also significantly more frequent with mazdutide. However, elevated lipase should not be equated with pancreatitis. Dulaglutide safety analyses have shown pancreatic enzyme elevations without a clear corresponding excess in adjudicated pancreatitis, and similar findings have been reported in Chinese populations [38, 39]. Accordingly, lipase elevation in our analysis is better interpreted as a biochemical monitoring signal rather than direct evidence of pancreatic injury, although it still supports the need for pancreatic enzyme surveillance in longer-term comparative studies.

Hypersensitivity, sinus tachycardia, and first-degree atrioventricular block did not differ significantly between groups. These null findings are still informative but should be interpreted cautiously because such events were uncommon and the confidence intervals were wide. Existing literature suggests that hypersensitivity reactions and heart-rate effects can occur with GLP-1 receptor agonists, including dulaglutide [40, 41]. Randomized trials are often underpowered to detect clear differences for these relatively rare outcomes [42]. Our findings therefore support the absence of a clear differential safety signal, rather than proving absence of risk.

### Limitations and strengths

This study has several limitations. The direct comparative evidence base remains small, limiting precision, particularly for rare safety outcomes. All three included trials were sponsored by the same manufacturer as part of a single clinical development program for mazdutide, and all were conducted exclusively in Chinese populations; this lack of independent replication and geographic diversity is an important limitation of external validity, and the findings may not generalize to other populations, healthcare settings, or sponsor contexts. In addition, all included head-to-head trials had relatively short follow-up, which may limit assessment of longer-term efficacy and tolerability. Notably, none of the included trials reported cardiovascular or renal outcomes, which represents a major limitation given that cardiovascular and renal protection are now central pillars of contemporary T2D management standards and a key consideration in the positioning of incretin-based therapies; the comparative cardiorenal profile of mazdutide versus dulaglutide therefore remains undefined and warrants dedicated investigation. Some outcomes, especially body-weight change and the strictest HbA1c thresholds, also showed notable heterogeneity in the primary analyses, although sensitivity analyses did not materially change the overall interpretation. Permitted background antidiabetic therapy also varied slightly across the included trials: two trials restricted background treatment to metformin alone (or diet and exercise), whereas the third additionally permitted metformin combined with an SGLT2 inhibitor or sulfonylurea in a subset of participants, representing a source of clinical heterogeneity not captured by our dose-based subgroup analyses and one that may be particularly relevant to outcomes such as hypoglycemia. In addition, hypoglycemia was not defined consistently across trials, two used the ADA classification (2013 and 2022, respectively) while the third reported it only as an unclassified adverse event term and this inconsistency in case definition was not adjusted for in the pooled analysis. Finally, while efficacy outcomes were generally at low risk of bias, safety outcomes had some concerns mainly related to outcome measurement, warranting greater caution in interpreting adverse-event comparisons.

These limitations are balanced by important strengths. This meta-analysis addressed a clinically meaningful head-to-head comparison against an established active comparator rather than placebo alone. Furthermore, we included only RCTs, which are considered the gold standard of study design. The efficacy findings were consistent across glycemic, weight, and composite outcomes, while the safety findings showed a coherent pattern of increased mainly gastrointestinal and non-serious adverse events without significant increases in serious adverse events or discontinuations. Taken together, these findings are consistent with, but given the small number of included trials do not definitively establish, the interpretation that mazdutide offers greater metabolic benefit, particularly for weight-related outcomes, at the cost of a higher burden of mostly non-serious adverse effects.

## Conclusion

Overall, based on three RCTs, the available evidence suggests that mazdutide may provide a meaningful advantage over dulaglutide in adults with type 2 diabetes, especially for body-weight reduction, clinically relevant weight-loss targets, and combined metabolic target attainment, while appearing to maintain a safety profile characterized mainly by increased non-serious adverse events. Given the small number of included trials, the single-sponsor and single-country origin of the evidence, and the short follow-up across all three studies, these conclusions should be regarded as preliminary rather than definitive. Larger, longer, and more diverse multinational trials are needed to confirm these findings, clarify durability of benefit, better define dose-response relationships, and determine the longer-term significance of signals such as hypoglycemia, lipase elevation, and infrequent cardiac or hypersensitivity events.

## Supplementary Information

Supplementary Material 1.

## Data Availability

No datasets were generated or analysed during the current study.

## Abbreviations

AE: Adverse Event
AV Block: Atrioventricular Block
BMI: Body Mass Index
CENTRAL: Cochrane Central Register of Controlled Trials
CI: Confidence Interval
DOI: Digital Object Identifier
FPG: Fasting Plasma Glucose
GLP-1: Glucagon-Like Peptide-1
GLP-1 RA: Glucagon-Like Peptide-1 Receptor Agonist
GRADE: Grading of Recommendations, Assessment, Development, and Evaluation
HbA1c: Glycated Hemoglobin A1c
MD: Mean Difference
MeSH: Medical Subject Headings
OR: Odds Ratio
PRISMA: Preferred Reporting Items for Systematic Reviews and Meta-Analyses
PROSPERO: International Prospective Register of Systematic Reviews
RCT: Randomized Controlled Trial
RoB 2: Cochrane Risk of Bias 2 Tool
RR: Relative Risk (or Risk Ratio)
SAE: Serious Adverse Event
T2DM: Type 2 Diabetes Mellitus
TEAE: Treatment-Emergent Adverse Event
