# Supplementary material for: Efficacy and safety of mazdutide vs. dulaglutide in adults with type 2 diabetes: a systematic review and meta-analysis of randomized controlled trials
Source: Diabetol Metab Syndr. 2026 Jul 24;18:219. doi: 10.1186/s13098-026-02248-3 (PMC13682033; doi:10.1186/s13098-026-02248-3)
Supplement: Supplementary file 1 [file 13098_2026_2248_MOESM1_ESM.docx]

| **Database** | **Search Query** | **Fields/Filters** | **Number of Results** |
| --- | --- | --- | --- |
| **Embase** | (mazdutide OR IBI362 OR LY3305677 OR dulaglutide OR Trulicity) AND ("type 2 diabetes" OR "type II diabetes" OR T2DM OR diabetes mellitus) | ti,ab | 651 |
| **Scopus** | (mazdutide OR IBI362 OR LY3305677 OR dulaglutide OR Trulicity) AND ("type 2 diabetes" OR "type II diabetes" OR T2DM OR diabetes mellitus) | TITLE-ABS | 344 |
| **PubMed** | (mazdutide[tiab] OR IBI362[tiab] OR LY3305677[tiab] OR dulaglutide[Mesh] OR dulaglutide[tiab] OR Trulicity[tiab]) AND ("Diabetes Mellitus, Type 2"[Mesh] OR "type 2 diabetes"[tiab] OR T2DM[tiab] OR diabetic*[tiab]) | tiab/MeSH | 128 |
| **Cochrane** | (mazdutide OR IBI362 OR LY3305677 OR dulaglutide OR Trulicity) AND ("type 2 diabetes" OR diabetic* OR T2DM) | All fields | 220 |

**Supplementary Table 1.** Search strategy

| **Outcome** | **Subgroup** | **Estimate [95% CI]** | **p-value** |
| --- | --- | --- | --- |
| **HbA1c Change (%)** | ≤ 20 Weeks | MD −0.16 [−0.47, 0.14] | 0.30 |
|  | > 20 Weeks | MD −0.27 [−0.42, −0.13] | 0.0002 |
| **Body Weight Change (Kg)** | ≤ 4mg | MD −2.27 [−3.85, −0.68] | 0.005 |
|  | > 4mg | MD −3.51 [−5.06, −1.96] | <0.00001 |
|  | ≤ 20 Weeks | MD −2.20 [−3.50, −0.91] | 0.0008 |
|  | > 20 Weeks | MD −3.70 [−5.32, −2.09] | <0.00001 |
| **Body Weight Change (%)** | ≤ 4mg | MD −2.54 [−4.66, −0.42] | 0.02 |
|  | > 4mg | MD −4.73 [−6.05, −3.41] | <0.00001 |
|  | ≤ 20 Weeks | MD −2.82 [−4.18, −1.45] | <0.0001 |
|  | > 20 Weeks | MD −4.77 [−6.71, −2.83] | <0.00001 |
| **Fasting Plasma Glucose (mmol/L)** | ≤ 4mg | MD 0.01 [−0.73, 0.75] | 0.97 |
|  | > 4mg | MD −0.23 [−0.56, 0.11] | 0.18 |
|  | ≤ 20 Weeks | MD −0.03 [−0.59, 0.54] | 0.93 |
|  | > 20 Weeks | MD −0.28 [−0.54, −0.01] | 0.04 |
| **HbA1c < 7.0%** | ≤ 4mg | RR 1.08 [0.92, 1.26] | 0.33 |
|  | > 4mg | RR 1.14 [0.99, 1.32] | 0.06 |
|  | ≤ 20 Weeks | RR 1.08 [0.84, 1.40] | 0.55 |
|  | > 20 Weeks | RR 1.12 [1.00, 1.26] | 0.05 |
| **HbA1c ≤ 6.5%** | ≤ 4mg | RR 0.93 [0.45, 1.93] | 0.85 |
|  | > 4mg | RR 1.38 [1.12, 1.69] | 0.002 |
|  | ≤ 20 Weeks | RR 0.97 [0.63, 1.48] | 0.87 |
|  | > 20 Weeks | RR 1.36 [1.15, 1.61] | 0.0004 |
| **HbA1c ≤ 5.7%** | ≤ 4mg | RR 1.66 [0.39, 7.08] | 0.49 |
|  | > 4mg | RR 1.90 [0.66, 5.47] | 0.24 |
|  | ≤ 20 Weeks | RR 0.82 [0.30, 2.20] | 0.69 |
|  | > 20 Weeks | RR 3.36 [1.76, 6.42] | 0.0002 |
| **≥5% Weight Loss** | ≤ 4mg | RR 2.10 [1.56, 2.83] | <0.00001 |
|  | > 4mg | RR 2.77 [2.08, 3.68] | <0.00001 |
|  | ≤ 20 Weeks | RR 2.11 [1.13, 3.96] | 0.02 |
|  | > 20 Weeks | RR 2.47 [1.92, 3.17] | <0.00001 |
| **≥10% Weight Loss** | ≤ 4mg | RR 7.25 [2.85, 18.42] | <0.0001 |
|  | > 4mg | RR 10.09 [4.20, 24.24] | <0.00001 |
|  | ≤ 20 Weeks | RR 5.70 [1.13, 28.87] | 0.04 |
|  | > 20 Weeks | RR 9.32 [4.65, 18.68] | <0.00001 |
| **≥5% Weight Loss + HbA1c <7.0%** | ≤ 4mg | RR 2.38 [1.63, 3.47] | <0.00001 |
|  | > 4mg | RR 3.19 [2.24, 4.55] | <0.00001 |
|  | ≤ 20 Weeks | RR 2.44 [1.10, 5.41] | 0.03 |
|  | > 20 Weeks | RR 2.82 [2.15, 3.71] | <0.00001 |

*MD = mean difference; RR = risk ratio.*

**Supplementary Table 2: Efficacy Outcomes (Subgroups)**

| **Outcome** | **Subgroup** | **Estimate [95% CI]** | **p-value** |
| --- | --- | --- | --- |
| **Any TEAEs** | ≤ 4mg | OR 1.87 [1.07, 3.29] | 0.03 |
|  | > 4mg | OR 1.58 [0.96, 2.61] | 0.07 |
|  | ≤ 20 Weeks | OR 1.44 [0.70, 2.94] | 0.32 |
|  | > 20 Weeks | OR 1.82 [1.17, 2.82] | 0.008 |
| **Serious Adverse Events** | ≤ 4mg | OR 0.72 [0.32, 1.62] | 0.43 |
|  | > 4mg | OR 0.51 [0.24, 1.10] | 0.09 |
|  | ≤ 20 Weeks | OR 0.35 [0.09, 1.34] | 0.13 |
|  | > 20 Weeks | OR 0.67 [0.36, 1.24] | 0.20 |
| **TEAEs Leading to Discontinuation** | ≤ 4mg | OR 0.55 [0.06, 5.46] | 0.61 |
|  | > 4mg | OR 0.44 [0.04, 4.49] | 0.49 |
|  | ≤ 20 Weeks | OR 0.11 [0.02, 0.70] | 0.02 |
|  | > 20 Weeks | OR 1.75 [0.56, 5.41] | 0.33 |
| **Diarrhea** | ≤ 4mg | OR 2.63 [1.61, 4.30] | 0.0001 |
|  | > 4mg | OR 2.27 [1.44, 3.59] | 0.0004 |
|  | ≤ 20 Weeks | OR 2.24 [1.05, 4.80] | 0.04 |
|  | > 20 Weeks | OR 2.48 [1.71, 3.60] | <0.00001 |
| **Decreased Appetite** | ≤ 4mg | OR 1.53 [0.55, 4.29] | 0.42 |
|  | > 4mg | OR 1.93 [1.24, 3.01] | 0.004 |
|  | ≤ 20 Weeks | OR 1.68 [0.80, 3.55] | 0.17 |
|  | > 20 Weeks | OR 1.96 [1.36, 2.81] | 0.0003 |
| **Nausea** | ≤ 4mg | OR 1.49 [0.50, 4.44] | 0.47 |
|  | > 4mg | OR 1.23 [0.56, 2.71] | 0.60 |
|  | ≤ 20 Weeks | OR 0.71 [0.36, 1.39] | 0.32 |
|  | > 20 Weeks | OR 2.60 [1.78, 3.81] | <0.00001 |
| **Vomiting** | ≤ 4mg | OR 1.10 [0.30, 3.97] | 0.89 |
|  | > 4mg | OR 1.37 [0.60, 3.12] | 0.46 |
|  | ≤ 20 Weeks | OR 0.74 [0.32, 1.72] | 0.48 |
|  | > 20 Weeks | OR 2.17 [1.44, 3.27] | 0.0002 |
| **Hypoglycemia** | ≤ 4mg | OR 1.62 [0.58, 4.52] | 0.36 |
|  | > 4mg | OR 1.86 [0.74, 4.70] | 0.19 |
|  | ≤ 20 Weeks | OR 2.39 [0.66, 8.65] | 0.18 |
|  | > 20 Weeks | OR 1.54 [0.68, 3.48] | 0.30 |
| **Hypersensitivity** | ≤ 4mg | OR 0.55 [0.02, 12.43] | 0.71 |
|  | > 4mg | OR 0.20 [0.03, 1.18] | 0.08 |
|  | ≤ 20 Weeks | OR 0.11 [0.02, 0.70] | 0.02 |
|  | > 20 Weeks | OR 1.05 [0.13, 8.19] | 0.96 |
| **Sinus Tachycardia** | ≤ 4mg | OR 1.78 [0.43, 7.31] | 0.42 |
|  | > 4mg | OR 0.40 [0.07, 2.41] | 0.32 |
|  | ≤ 20 Weeks | OR 0.29 [0.05, 1.89] | 0.20 |
|  | > 20 Weeks | OR 1.51 [0.48, 4.76] | 0.48 |
| **Abdominal Distension** | ≤ 4mg | OR 1.42 [0.73, 2.78] | 0.30 |
|  | > 4mg | OR 1.12 [0.61, 2.06] | 0.72 |
|  | ≤ 20 Weeks | OR 0.88 [0.30, 2.57] | 0.81 |
|  | > 20 Weeks | OR 1.35 [0.82, 2.22] | 0.24 |
| **AV 1st Degree Block** | ≤ 4mg | OR 0.11 [0.00, 2.75] | 0.18 |
|  | > 4mg | OR 0.59 [0.09, 3.91] | 0.58 |
| **Lipase Levels** | ≤ 4mg | OR 2.11 [0.77, 5.76] | 0.15 |
|  | > 4mg | OR 3.48 [1.25, 9.69] | 0.02 |
|  | ≤ 20 Weeks | OR 1.92 [0.07, 55.84] | 0.70 |
|  | > 20 Weeks | OR 2.74 [1.32, 5.71] | 0.007 |

**Supplementary Table 3: Adverse Events (Subgroups)**

| **Outcome** | **Type** | **Effect Estimate** | **I²** | **Risk of Bias** | **Inconsistency** | **Indirectness** | **Imprecision** | **Publication Bias** | **GRADE Certainty** |
| --- | --- | --- | --- | --- | --- | --- | --- | --- | --- |
| **HbA1c Change (%)** | Continuous | MD −0.25 [−0.38, −0.12] | 0% | Not serious | Not serious | Not serious | Not serious | Undetected | HIGH |
| **Body Weight (kg)** | Continuous | MD −2.97 [−4.14, −1.80] | 74% | Not serious | Very seriousᵃ | Not serious | Not serious | Undetected | LOW |
| **Body Weight (%)** | Continuous | MD −3.73 [−5.02, −2.43] | 56% | Not serious | Very seriousᵃ | Not serious | Not serious | Undetected | LOW |
| **FPG (mmol/L)** | Continuous | MD −0.23 [−0.47, 0.01] | 0% | Not serious | Not serious | Not serious | Seriousᵇ | Undetected | MODERATE |
| **HbA1c <7.0%** | Dichotomous | RR 1.11 [1.00, 1.24] | 0% | Not serious | Not serious | Not serious | Not serious | Undetected | HIGH |
| **HbA1c ≤6.5%** | Dichotomous | RR 1.22 [0.99, 1.52] | 35% | Not serious | Not serious | Not serious | Seriousᵇ | Undetected | MODERATE |
| **HbA1c ≤5.7%** | Dichotomous | RR 1.95 [0.97, 3.90] | 31% | Not serious | Not serious | Not serious | Seriousᵇ | Undetected | MODERATE |
| **≥5% Weight Loss** | Dichotomous | RR 2.43 [1.98, 2.98] | 0% | Not serious | Not serious | Not serious | Not serious | Undetected | HIGH |
| **≥10% Weight Loss** | Dichotomous | RR 8.64 [4.56, 16.37] | 0% | Not serious | Not serious | Not serious | Not serious | Undetected | HIGH |
| **≥5% Weight Loss + HbA1c <7.0%** | Dichotomous | RR 2.78 [2.15, 3.60] | 0% | Not serious | Not serious | Not serious | Not serious | Undetected | HIGH |
| **Any TEAEs** | Safety | OR 1.70 [1.17, 2.48] | 0% | Not serious | Not serious | Not serious | Not serious | Undetected | HIGH |
| **Serious Adverse Events** | Safety | OR 0.60 [0.34, 1.05] | 0% | Not serious | Not serious | Not serious | Seriousᵇ | Undetected | MODERATE |
| **TEAEs Leading to Treatment Discontinuation** | Safety | OR 0.59 [0.15, 2.24] | 39% | Not serious | Not serious | Not serious | Very seriousᶜ | Undetected | LOW |
| **Diarrhea** | Safety | OR 2.43 [1.74, 3.40] | 0% | Not serious | Not serious | Not serious | Not serious | Undetected | HIGH |
| **Decreased Appetite** | Safety | OR 1.90 [1.37, 2.63] | 0% | Not serious | Not serious | Not serious | Not serious | Undetected | HIGH |
| **Nausea** | Safety | OR 1.48 [0.88, 2.48] | 38% | Not serious | Not serious | Not serious | Seriousᵇ | Undetected | MODERATE |
| **Vomiting** | Safety | OR 1.48 [0.87, 2.53] | 27% | Not serious | Not serious | Not serious | Seriousᵇ | Undetected | MODERATE |
| **Hypoglycemia** | Safety | OR 1.75 [0.88, 3.48] | 0% | Not serious | Not serious | Not serious | Seriousᵇ | Undetected | MODERATE |
| **Hypersensitivity** | Safety | OR 0.30 [0.08, 1.21] | 0% | Not serious | Not serious | Not serious | Very seriousᶜ | Undetected | LOW |
| **Sinus Tachycardia** | Safety | OR 0.92 [0.33, 2.59] | 8% | Not serious | Not serious | Not serious | Seriousᶜ | Undetected | LOW |
| **Abdominal Distension** | Safety | OR 1.25 [0.79, 1.96] | 0% | Not serious | Not serious | Not serious | Seriousᵇ | Undetected | MODERATE |
| **AV 1st Degree Block** | Safety | OR 0.38 [0.07, 1.95] | 0% | Not serious | Not serious | Not serious | Very seriousᶜ | Undetected | LOW |
| **Increased Lipase** | Safety | OR 2.70 [1.32, 5.53] | 0% | Not serious | Not serious | Not serious | Not serious | Undetected | HIGH |

**Footnotes**

ᵃ Inconsistency: Downgraded due to substantial statistical heterogeneity (I² ≥50%), suggesting variability in effect magnitude across trials.

ᵇ Imprecision: Downgraded because the 95% confidence interval crossed the line of no effect or approached clinically important decision thresholds.

ᶜ Very serious imprecision: Downgraded by two levels due to sparse events, wide confidence intervals, or unstable estimates.

**Supplementary Table 4.** Grade Assessment.

Grades assigned based on predefined criteria to classify severity or extent according to study methodology.

**Supplementary Supplementary Figure 1: Summary of Risk of Bias Assessment for Safety Outcomes**
